# P86 - Magnesium sulphate in the management of severe asthma and atelectasis

**DOI:** 10.1186/2045-7022-4-S1-P141

**Published:** 2014-02-28

**Authors:** Davendralingam Sinniah

**Affiliations:** 1 International Medical University Clinical School Seremban, Seremban, Malaysia

## Introduction

Magnesium sulphate (MS), causes bronchodilation by inhibiting bronchial smooth muscle contraction, interferes with parasympathetic stimulation, and prevents acetylcholine release to axon terminals [1]. It reduces inflammation by inhibiting mast cell degranulation and reduces thromboxane, histamine and leukotrienes [2]. Some pediatric studies suggest that MS, b-2-agonists and steroids are beneficial in acute moderate/severe asthma by reducing hospitalization and absolute risk [3].

## Objectives

This paper 1) reviews the randomized controlled trials in the literature on use of MS in asthma and 2) reports the dramatic resolution of massive pulmonary atelectasis in an asthmatic child within 2 hours of IV MS.

## Case Report

A 5-year-old boy with past asthma presented with rapidly progressing asthma unrelieved by prednisolone and multiple doses of salbutamol, ipratropium bromide. Examination revealed tachypnea, tachycardia, chest recession, tracheal tug, right tracheal deviation, dullness and decreased breath sounds in right lower chest and wheezing in other areas. Percutaneous Spo2 was 86% (room air) and 95% with oxygen 5 L/minute. WBC 27.3 x 10^9/L, neutrophils 25.7 x 10^9/L, lymphocytes 1.1 x19^9/L. CRP was 11.6 (<10mg/L). Chest x-ray confirmed atelectasis of right middle and lower lobes. Antibiotics were started followed by IV methylprednisone (1 mg/kg), aminophylline (10 mg/kg bolus), and MS (50 mg/kg). Chest findings normalized within 2 hours. Spo2 improved to 95% on 2 L oxygen/minute. Medication was discontinued save for salbutamol PRN, oral prednisolone (1 mg/kg/day), and MDI fluticasone 50 mcg (BD). On day 2, the child was active and playful. Chest examination and repeat chest x-ray were normal.

## Conclusion

Review of literature and dramatic resolution of asthma-related massive atelectasis following intravenous MS in our case establishes MS as an adjunct to standard therapy in patients with severe, acute asthma including atelectasis.

